# Anonymity Versus Privacy: Selective Information Sharing in Online Cancer Communities

**DOI:** 10.2196/jmir.2684

**Published:** 2014-05-14

**Authors:** Jeana Frost, Ivar E Vermeulen, Nienke Beekers

**Affiliations:** ^1^VU University AmsterdamAmsterdamNetherlands; ^2^National IT Institute for HealthcareAmsterdamNetherlands

**Keywords:** online systems, cancer, privacy, confidentiality, Health 2.0, anonymity

## Abstract

**Background:**

Active sharing in online cancer communities benefits patients. However, many patients refrain from sharing health information online due to privacy concerns. Existing research on privacy emphasizes data security and confidentiality, largely focusing on electronic medical records. Patient preferences around information sharing in online communities remain poorly understood. Consistent with the privacy calculus perspective adopted from e-commerce research, we suggest that patients approach online information sharing instrumentally, weighing privacy costs against participation benefits when deciding whether to share certain information. Consequently, we argue that patients prefer sharing clinical information over daily life and identity information that potentially compromises anonymity. Furthermore, we explore whether patients’ prior experiences, age, health, and gender affect perceived privacy costs and thus willingness to share information.

**Objective:**

The goal of the present study is to document patient preferences for sharing information within online health platforms.

**Methods:**

A total of 115 cancer patients reported sharing intentions for 15 different types of information, demographics, health status, prior privacy experiences, expected community utility, and privacy concerns.

**Results:**

Factor analysis on the 15 information types revealed 3 factors coinciding with 3 proposed information categories: clinical, daily life, and identity information. A within-subject ANOVA showed a strong preference for sharing clinical information compared to daily life and identity information (*F*_1,114_=135.59, *P*=.001, η^2^=.93). Also, adverse online privacy experiences, age, and health status negatively affected information-sharing intentions. Female patients shared information less willingly.

**Conclusions:**

Respondents’ information-sharing intentions depend on dispositional and situational factors. Patients share medical details more willingly than daily life or identity information. The results suggest the need to focus on anonymity rather than privacy in online communities.

## Introduction

### Overview

Sharing information through patient platforms offers new opportunities for patients to learn about and manage their condition. However, information sharing online also introduces risks to patient privacy. There is a growing interdisciplinary scholarship around privacy that seeks to define the concept and construct systems to securely move data through a network. But, to date, there is a lack of research on user preferences concerning privacy and information sharing [1]. Building on previous work in the area of information systems and sharing [2-4], we propose and test a model for how patients think about sharing information online. We suggest that patients will be most likely to share information when the benefits of doing so outweigh the risks. This cost benefit analysis is dynamic and varies according to who is sharing and the context of the exchange. Therefore, we argue that sharing preferences are best thought of in relational terms as a product of 3 factors: individual characteristics, type of information, and the breadth of the audience.

In this study, we look at the case of cancer patients and we distinguish 3 types of information: clinical, daily life, and identity information. We argue that although clinical information is “sensitive,” it is most relevant to online health discussions and, therefore, it is shared most easily. Daily life and identity information are often shared in face-to-face conversations, but impose greater risk to anonymity and are less relevant for online health discussions. Therefore, they are shared less broadly. Individual factors further impact willingness to share both generally and around specific information types.

### Background

Cancer rates are increasing across the developed world. In America, the lifetime risk of developing cancer is now 1 in 2 for men and 1 in 3 for women [5]. As the population in America and Europe ages, the cancer burden is expected to increase [6].

Cancer patients have a set of unmet psychosocial and informational needs that change over the course of treatment (for a review [7]). Although treatment needs near the time of diagnosis are often met, long-term physical, psychological, and psychosocial problems are given less attention [8]. The most frequently reported unmet needs of patients include psychological support [9,10], managing practical problems related to daily living, fear of recurrence [9], and information about genetics and the disease itself [11]. Studies also suggest the importance of addressing quality of life issues, both for improving survival rates (eg, [12]) and as a positive outcome measure [13,14]. For many of these unmet needs, there is no clear course of treatment or a “quick fix.” Rather, patients and their families must learn to adapt, cope, and manage the variety of issues that arise.

**Figure 1:**
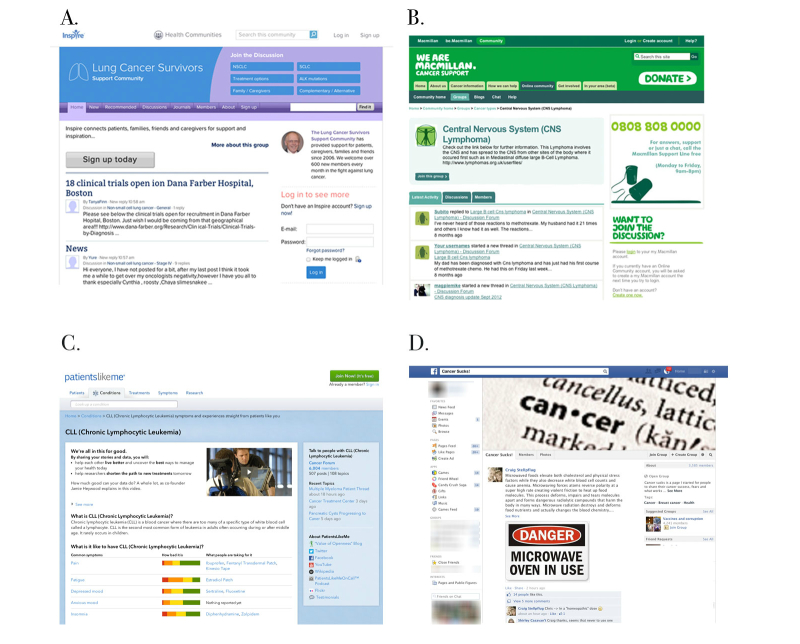
Screenshots of various Web platforms available for peer-to-peer discussion of cancer, including general platforms for many types of conditions including cancer (A and C), platforms for cancer specifically (B), and social media (D).

Websites and Health 2.0 platforms are well-positioned to address these unmet patient needs. A growing set of engaged people, or ePatients, go online to search for information and connect with one another by exchanging health information and social support [15,16] on a variety of platforms (see Figure 1). Although findings are still mixed [17], a nascent body of literature suggests that these exchanges help address the otherwise unmet needs of cancer patients. In general, peer-to-peer support provides informational, emotional, and instrumental benefits [18]. For cancer patients specifically, online communities help in a variety of ways, including providing information on treatment and how to communicate with physicians, and emotional support on how to cope with cancer [19,20]. Despite some concern on the part of physicians, involvement in online communities seems to complement rather than replace the information and support coming from professionals. Indeed, research suggests that peers provide qualitatively different information [21] and support [22] than medical experts. Online communities are available anywhere, anytime, and potentially provide a place where sensitive topics can be safely discussed. In fact, people with sensitive problems who might have difficulty discussing these issues face-to-face are more likely to participate in online communities than people with conditions that are not stigmatized [23].

Active participation on online platforms appears to benefit both individual members and the community at large. Although passive viewing, or lurking, in an online community appears to be helpful for cancer patients [24], active participation in online communities has been linked to positive outcomes both online and offline, including improved mood [25], greater perceived online support, and offline improvements [26]. Perhaps more importantly, the value of a platform is tied to the level of user-generated content. Through sharing information and insight online, active participants improve the quality of the community. Therefore, it is important to promote active information sharing for the good of the individual members and the community at large.

Although Health 2.0 platforms present opportunities for patients, they also introduce the possibility of privacy invasions that could result in prejudice, decreases in economic opportunity, and potentially a loss of health care coverage. Past research suggests that concerns about privacy translate into online behavior—privacy concerns remain a key barrier to sharing information in online communities (eg, [27-29]). And privacy is a primary reason people cite for simply collecting information rather than actively participating online [30].

Despite the importance of privacy within online patient platforms and the Internet in general, users’ preferences around information sharing are not well understood. Although online privacy is a rich interdisciplinary area of research, a literature review reveals a lack of attention to users’ perspective in the design of privacy tools [1]. A similar gap exists in health care. Health care privacy research tends to treat privacy as a single construct, with an emphasis on protecting patient information to facilitate online exchanges. Most health care privacy research focuses implicitly or explicitly on data security within clinical systems, such as online electronic medical records and personal health records for which there are both moral and legal obligations to guard users from unintended harm (for a review [31]). The existing research on patient perspectives on privacy focuses on individual differences in willingness to share information [27,32], thereby, implicitly treating all types of information equally.

Legal and information systems research suggests that patient preferences might be complex. Legal scholars, noting an oversimplified use of the word “privacy” from the individual’s perspective, have called for a more nuanced view—one that acknowledges the variety of types of invasions to privacy and examines the significance of privacy within a particular situation [33]. For the purpose of this study, we use Westin’s concept of privacy: the right to privacy is the individual’s ability to determine when, how, and to what extent information can be shared [34]. Related to the current work, research on consumers’ willingness to disclose personal information on retail sites depends upon the nature of the information [35]. Building upon this legal and privacy scholarship, we propose that preferences around sharing information in online health platforms are not uniform; rather, they vary with types of information and individual factors.

In particular, preferences may differ depending upon the perceived benefit of sharing a particular type of information with the anticipated audience. Within the context of e-commerce, people seem to conduct a mental calculation weighing the benefits against the costs of disclosing personal information to the system before making a purchase; this mental calculation has been labeled the “privacy calculus” [2]. In the health care domain, research suggests that patients choose to share information in situations when the expected value of sharing outweighs the possible risks [29].

In the current study, we will focus on 3 types of information sharing: clinical, daily life, and identity information. By clinical information, we mean detailed medical data describing diagnosis history, treatments, symptoms, and outcomes (eg, diagnosis data, cancer type, treatment regimen). We suggest that clinical information is pertinent to medical discussions and provides benefits for the online experience. When clinical information is shared without disclosing identity information (described subsequently), clinical information imposes low privacy costs. By daily life information, we mean information about professional life and relationships (eg, marital status and occupation). Although people may routinely share such information in casual face-to-face conversations, daily life information has marginal benefits for health conversations while introducing intermediate privacy costs. Finally, by identity information we mean information (eg, photo or personal email address) with little relevance in discussions about patient knowledge and psychological well-being, yet imposing high privacy costs—especially in combination with disclosed clinical information—by compromising user anonymity. Therefore, when viewed from the calculation of users weighing psychosocial and medical benefits against privacy costs, we expect that although some pieces of information may be relevant to more than one category, clinical information will be most easily shared, followed by daily life information, and then identity information.

In addition, we explore how personal characteristics and previous experiences online affect intentions around information sharing. First, intentions to share information may positively relate to the value patients anticipate from using a particular system and negatively relate to individuals’ privacy concerns. Second, patients who have the highest expectation of life after cancer (eg, patients who are younger and have a better prognosis) may be the most reluctant to share health information [36]. Third, several studies suggest that women perceive higher online privacy costs than men [3,37,38], also in the context of health-related information [39]. Consequently, women may be less willing to share identity information than men.

In this study, we test patient preferences around privacy and anonymity with the central argument that people will be more interested in sharing information that is the stated topic of the online community. Furthermore, we expect that the willingness to share information depends on a combination of dispositional and situational factors [32]. To evaluate these hypotheses, we survey cancer patients interested in joining an online cancer community. Patients report on demographics, health status, expected utility of the forum, general privacy concerns, as well as on their willingness to share different types of information with different size audiences.

## Methods

### Participants

**Figure 2:**
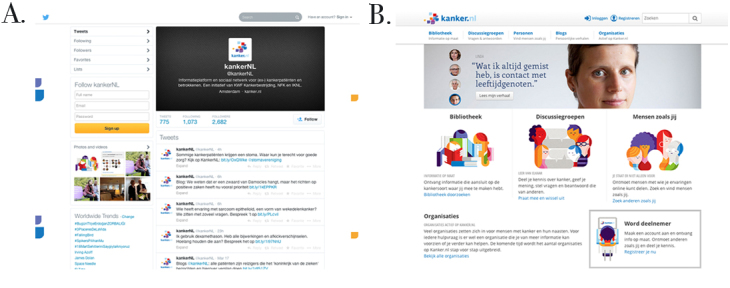
Screenshots of the Kanker.nl Twitter feed (A) and the subsequently released Kanker.nl home page (B).

We recruited respondents through a website created to inform the design of a Dutch patient platform for cancer patients, Kanker.nl [40], and the platform’s Twitter feed (see Figure 2). In total, 132 people completed the survey; 17 nonpatients were excluded from the analysis, leaving 115 current or previous cancer patients in the sample. The survey included the measures reported subsequently as well as items on the desired features of the Kanker.nl platform.

Table 1 displays the sample’s characteristics. Participants had been diagnosed with a variety of cancer types, but 3 cancer groups were most prevalent: leukemia, bone marrow, and lymphoma (39.1%, 45/115); breast cancer (17.4%, 20/115); and cancers that affect the digestive organs (9.6%, 11/115). On average, the patients in the sample were diagnosed 6 (SD 6) years before the survey was conducted in 2006. Of all participants, 40.9% (47/115) were currently in treatment, 56.5% (65/115) had completed treatment, and 1.7% (2/115) could no longer be treated. The sample included more women (54.8%, 63/115) than men, and ranged in age (mean 52, SD 12, range 20-75).

**Table 1:** Baseline characteristics of the respondents (N=115).

Demographic variable		Response
**Sex, n (%)**		
	Women	63 (54.8)
	Men	52 (45.2)
Age (years), mean (SD)		52 (12)
Years since diagnosis, mean (SD)		6 (6)
**Most commonly reported cancer types, n (%)**		
	Leukemia, bone marrow, and lymphoma	45 (39.1)
	Breast cancer	20 (17.4)
	Cancers that affected the digestive organs and	11 (9.6)
**Treatment status, n (%)**		
	In treatment	47 (40.9)
	Within 1 year of treatment	21 (18.2)
	With 5 years of treatment	23 (20.0)
	More than 5 years post treatment	21 (18.3)
	Not treatable	2 (1.7)

### Instrument Development

The study employs a combination of existing and self-developed items (see Table 2). All items were translated into Dutch and entered into a Likert scale of 1 to 7, unless otherwise specified. Based on previous research demonstrating the reproducibility, reliability, and performance of a single item health status measure [28], we posed a single question asking respondents to rate their health status (“In general my health is...” with 1=very poor to 10=very good). The perceived usefulness of the future community was measured by using a question adapted from previous work (“How useful do you expect Kanker.nl will be for you?” with responses ranging from not at all to very useful) [29,21]. Health information privacy concerns were measured using 2 items (alpha=.84): (1) “I believe that submitting health information on the Internet is...” with responses ranging from not advisable at all to highly advisable and (2) “Health information on the Internet, once submitted...” with responses ranging from will not be misused at all to will be misused for sure [29]. Prior negative experiences with information sharing were measured using 1 item adapted from work on trust and information sources (“When it comes to the privacy invasion of health information, my online experience could be characterized as...” with responses ranging from no bad experiences to very bad experiences) [41]. Finally, patients reported on their phase of treatment by choosing between a closed set of options.

**Table 2:** Sources and text for all items except demographic variables and treatment phase (translated from Dutch).

Construct	Source	Item(s)	Response options
Health status	[42]	Overall, my health status is...	Poor to very good
Expected utility of the platform	[29,41]	How useful do you think kanker.nl will be for you?	Not at all to very useful
Prior negative experience	[23]	My experiences with privacy infringement on personal health can be described as...	Not at all negative to very negative
Privacy concern	[23]	I believe that submitting health information on the Internet is...	Highly advisable/not advisable at all
		Health information on the Internet, once submitted...	Will not be abused at all/will be abused for sure
Intention to share information	Self-developed	We are currently designing the privacy settings for Kanker.nl, for each piece of information, please indicate which group you would like to share it with	Only with personal contacts/only with members of the website/with all website visitors

To assess intentions to share different types of information, we asked participants about the breadth of the audience with whom they would like to share. To approximate the real world setting being studied, these questions were asked as if the respondents were choosing privacy settings for an online community. This community was described as a platform providing both expert and patient-generated information as well as peer-to-peer communication about cancer. Items were piloted in focus groups for clarity [27]. The response categories ranged from a smaller to larger audience, including (1) personal contacts, (2) members of the site, or (3) all website visitors. Participants were asked for sharing intentions with respect to 15 types of information: sex, age, marital status, family situation, profession, place of residence, province, picture, email address, patient status (ie, labeled as patient), type of cancer, date of diagnosis, treatment status, hospital, and clinician.

Using SPSS version 20 (IBM Corp, Armonk, NY, USA), we conducted a varimax-rotated principal component analysis (PCA), a variable reduction technique similar to factor analysis, to cluster together highly correlating items out of the 15 assessed. The PCA produced 3 factors with eigenvalues >1 together accounting for 73.05% of the variance (factor 1: 50.48%; factor 2: 13.34%; factor 3: 9.24%). Items were retained that loaded higher than 0.3 on their primary factor and had a primary loading of at least 0.2 higher than any of their cross-loadings.

Items pertaining to sharing clinical information (patient status, type of cancer, date of diagnosis, treatment status, sex, and age) loaded strongly on the first factor and were clustered together in a scale (alpha=.94). Items relating to sharing information about daily life (marital status, family situation, profession, and county) loaded strongly on the second factor (scale: alpha=.90). Items pertaining to sharing identity information (place of residence, picture, email address) loaded strongly on the third factor (scale: alpha=.78). Two items pertaining to sharing information about specific hospitals and clinicians loaded similarly on both the clinical and the identity dimension; thus, they were not included in either scale. In addition to the 3 subscales, we constructed a scale for overall sharing intentions of all 15 items (alpha=.93).

## Results

A within-subject ANOVA showed that, consistent with our expectations, clinical information was most broadly shared (mean 2.32, SD 0.60, scale range 1-3), followed by daily life information (mean 1.86, SD 0.66), and identity information (mean 1.58, SD 0.56). The overall difference in intentions to share the 3 types of information was significant (*F*_1,114_=135.59, *P*<.001, η^2^=.93), as were the pairwise differences (all *P*s<.001). See Table 3 for item-based responses.

**Table 3:** Willingness to share items: frequencies, means, and standard deviations.

Type of information		Willingness to share, n (%)			Mean (SD)
		Only with personal contacts	Only with members of the website	With all website visitors	
**Clinical information**					2.32 (0.60)
	Patient status	11 (9.6)	49 (42.6)	55 (47.8)	2.38 (0.66)
	Type of cancer	9 (7.8)	44 (38.3)	62 (53.9)	2.46 (0.64)
	Date of diagnosis	15 (13.0)	61 (53.0)	39 (33.9)	2.21 (0.66)
	Treatment status	17 (14.8)	58 (50.4)	40 (34.8)	2.20 (0.68)
	Sex	12 (10.4)	44 (38.3)	59 (51.3)	2.41 (0.67)
	Age	21 (18.3)	44 (38.3)	50 (43.5)	2.25 (0.75)
**Daily life information**					1.86 (0.67)
	Marital status	50 (43.5)	38 (33.0)	27 (23.5)	1.80 (0.80)
	Family situation	46 (40.0)	46 (40.0)	23 (20.0)	1.80 (0.75)
	Profession	46 (40.0)	43 (37.4)	26 (22.6)	1.83 (0.78)
	County	29 (25.2)	57 (49.6)	29 (25.2)	2.00 (0.71)
**Identity information**					1.58 (0.56)
	Place of residence	52 (45.2)	49 (42.6)	14 (12.2)	1.67 (0.68)
	Picture	65 (56.6)	36 (31.3)	14 (12.2)	1.56 (0.70)
	Email address	66 (57.4)	40 (34.8)	9 (7.8)	1.50 (0.64)
**Noncategorized information**					
	Specific hospital	20 (17.4)	63 (54.8)	32 (27.8)	2.10 (0.67)
	Specific clinician	35 (30.4)	57 (49.6)	23 (20.0)	1.90 (0.70)

Table 4 shows the effects of individual differences on general sharing intentions. The model includes variables previously associated with differences in sharing preferences: expected value of the overall platform, general privacy concerns, health status, sex, age, and negative experiences online. Contrary to expectations, expected value of the platform, general privacy concerns, and sex (added as a dummy: male=–1, female=1) did not significantly predict general sharing intentions. However, results showed that prior negative experiences online (beta=–.43, *P*<.001) had a strong negative effect on sharing intentions. Also, older patients shared information more broadly than younger patients did (beta=.11, *P*=.01), and patients’ health status had a marginal negative effect on general sharing intentions (beta=–.15, *P*=.07). The total regression model explained 25% (*R*=.54, adjusted *R* ^*2*^=.25) of the variance in intentions to share identity information (*F*_5,109_=7.42, *P*<.001).

**Table 4:** Regression analysis: effects of interpersonal differences on cancer patients’ general information-sharing intentions on an online platform.

Predictors	B	Beta	*t*_113_	*P*
Expected utility of platform	.04	.08	0.99	.32
General privacy concerns	–.05	–.10	–1.14	.26
Negative experiences with online privacy	–.21	–.43	–4.97	<.001
Age	.11	.22	2.63	.01
Health status	–.07	–.15	–1.81	.07
Sex (dummy)	.30	.28	2.56	.01

Table 5 focuses on the effects of individual differences on intentions to share identity information. Contrary to expectations, the expected value of the platform did not produce the predicted positive effect. However, the effect of general privacy concerns (beta=–.19, *P*=.047) and adverse experiences with online privacy (beta=–.24, *P*=.008) negatively impacted sharing intentions as we expected. Also, older patients (beta=.24, *P*=.008) and patients with poorer health status (beta=–.26, *P*=.003) had fewer problems disclosing identity information, as did men (beta=–.19, *P*=.03). The total regression model explained 20% (*R*=.49, adjusted *R* ^*2*^=.20) of the variance in intentions to share identity information (*F*_5,109_= 5.67, *P*<.001).

Finally, an exploratory *t* test showed no differences in sharing intentions for any information category between patients who were either pretreatment or during treatment versus posttreatment (all *t*s<1.24).

**Table 5:** Regression analysis: effects of interpersonal differences on cancer patients’ intentions to share identity information on an online platform.

Predictors	B	Beta	*t*_113_	*P*
Expected utility of platform	.01	.02	0.25	.81
General privacy concerns	–.08	–.19	–2.01	.047
Negative experiences with online privacy	–.14	–.24	–2.68	.008
Age	.14	.24	2.72	.008
Health status	–.15	–.26	–3.03	.003
Sex (dummy)	–.11	.19	–2.17	.03

## Discussion

### Principal Findings

This work suggests that privacy concerns are not uniform and depend on both individual and contextual factors. The privacy calculus model argues that people share information if the perceived rewards outweigh the perceived risks; as a result, people are selectively willing to share different types of information [2-4]. Although the selectivity in information sharing may be intuitive, the pattern of results is not. In the domain of patient platforms, the mental calculation about costs and benefits differs from other domains of life. During face-to-face conversations and in the context of generic online social platforms, such as Facebook, people routinely exchange names, ages, marital statuses, and professions. Yet, in the online health environment, ePatients are more willing to share what is often thought to be more sensitive health information. Platform members log on to share expertise based on medical experiences—details about their medical history and treatment are central to the experience. Indeed, although information may fit into more than 1 of the 3 identified domains, this study finds that patients are more willing to share clinical information than other forms of demographic and daily life information. Although patients mentioned in interviews that they would like to share daily life experiences with peers [36], the benefits of actively sharing such information with others appears lower than the benefits gained from sharing clinical information. As such, the privacy costs of sharing daily life information may often outweigh the possible benefits. In the case of identity information, risks of losing anonymity exceed the possible gains in the user experience and willingness to share is correspondingly low.

Our findings are consistent with previous research on the impact of prior experiences on willingness to share information [29] and sex [28]. Prior negative experiences regarding online privacy have a strong negative effect on patients’ intentions to share all different types of information. Female respondents were less willing to share identity information than their male counterparts were. In general, women comprise the majority of social network users and tend to actively participate in online communities at a greater rate than men [43,44]. Yet, in this study and in previous studies, women seem to be more concerned with being identified than men. As a result, women are less willing to share information that potentially compromises anonymity [28].

An interesting finding is that patients who may be more concerned with their “life after cancer” (ie, younger patients and patients with a better health status) are less willing to share information with peers. This finding suggests that patients are fairly pragmatic in assessing the risks involved with disclosing information online. Although younger people tend to seek more information when making health-related decisions [45] and, therefore, may benefit more from an online community, the risks that sharing information imposes on future opportunities and possibly higher Internet literacy—including knowledge about privacy-related issues—may prevent younger and healthier patients from doing so.

Our results diverge from previous findings on the relationship between the expected value of the online platform on sharing intentions. This outcome suggests that patients do not associate sharing personal information with general site benefits, such as receiving more tailored peer feedback on their specific condition or situation. Instead, when contemplating which information to share with whom, patients may think about the value of that information for specific exchanges or the benefit of sharing that information for peers; that is, they could be considering to what extent specific types of information may benefit a particular online conversation or other community members. Also, our results showed that general privacy concerns hamper sharing intentions only with respect to the specific category of identity information and not with respect to general sharing intentions. This outcome further supports the notion that all information sharing is not equal.

### Limitations

In this study, we examined intentions and preferences around sharing information within an online patient community through a survey. A basic limitation of our approach is the hypothetical nature of the questions asked. Respondents were recruited from a group of people interested in joining an online patient platform and the questions about privacy resembled those asked in an online community. However, our survey was conducted outside the context of such an online community, and asked for potential users’ sharing intentions rather than measuring actual sharing behaviors. In other settings, people routinely deviate from stated privacy preferences in their actual behavior [46]. Although patients may perform a cost benefit analysis when they are thoughtfully contemplating privacy settings in a survey, people may behave differently in actual online communities.

A second limitation to the study concerns the sampling of respondents. The respondents were members of an online panel of people interested in joining a new patient platform and volunteered for the study. As such, these particular individuals may have a higher level of Internet literacy than the patient population at large. Still, this sample seems representative of the population likely to participate in current and emerging online communities; indeed, most of our respondents expressed a desire to participate in the Kanker.nl platform.

Future work could examine the role of additional factors that influence sharing intentions and study sharing behavior directly. Emotional drivers (eg, fear, shame) may impact willingness to share within a peer-to-peer environment [47], complementing the more rational cost-benefit approach presented in the current research. Prosocial behavior and willingness to disclose information is associated with peers who do the same [48]. Through this process of disclosure, norms of trust may emerge. Consequently, future research could also look into reciprocity as a driver of willingness to share.

### Conclusions

The current study extends our understanding of patient privacy preferences by disaggregating the notion of privacy concerns. Different types of information pose different concerns, and different users have different concerns. Because of continual changes in technology and the user base of online health communities, understanding privacy concerns and sharing behaviors from a patient perspective is an ongoing process. However, such understanding is crucial to optimize the positive effects online health communities have on well-being.

This work has implications for designing online communities. Research shows that in many patient populations (eg, posttreatment patients) there are clear benefits to reading and reviewing content from online communities [24]. Several types of Health 2.0 platforms and communities now exist, some of which protect identity to such an extent that patients can only log on individually to access medical records, maintaining confidentiality (eg, Kaiser Permanente’s My Health Manager and the Mayo Clinic’s Patient Online Services), whereas others piggyback on existing social networks with no pretense of anonymity (eg, Genentech’s Circle of Support App for breast cancer patients and the Cancer Sucks! community on Facebook). The current research suggests an intermediate solution. To align with patient preferences, systems should guard anonymity while facilitating clinical information sharing.

## Abbreviations

PCA: principal component analysis
